# Effectiveness and Adherence Rate of S-flurbiprofen Plaster for the Pain Management of Patients With Moderate and End-Stage Knee Osteoarthritis

**DOI:** 10.7759/cureus.44556

**Published:** 2023-09-02

**Authors:** Masaki Iguchi, Tsuneari Takahashi, Katsushi Takeshita

**Affiliations:** 1 Department of Orthopaedic Surgery, Miyazaki Prefectural Nobeoka Hospital, Nobeoka, JPN; 2 Department of Orthopaedic Surgery, Ishibashi General Hospital, Shimotsuke, JPN; 3 Department of Orthopaedics, Jichi Medical University, Shimotsuke, JPN

**Keywords:** s-flurbiprofen plaster, adherence, side effect, nonsteroidal anti-inflammatory agents, s-flurbiprofen, non-surgical treatment, knee osteoarthritis

## Abstract

Background: S-flurbiprofen plaster (SFPP) is highly skin permeable and represents a new conservative treatment for knee osteoarthritis (KOA) that can attain considerably higher concentrations in the synovium than topical flurbiprofen. To date, no study has investigated the efficacy and adherence rate of SFPP in patients with end-stage KOA. This study aimed to compare the effectiveness and adherence rate of SFPP for pain management in patients with moderate and end-stage KOA.

Methods: This retrospective study included a total of 118 patients with KOA (Kellgren-Lawrence classification grades II (n = 29), III (n = 32), and IV (n = 57)). The difference in SFPP use rate, adverse drug reactions rate, whether 50% pain relief occurred, and the percentage of patients who underwent surgical treatment were calculated.

Results: The overall SFPP use rate at one year was 61.0% (88.1% at less than one month, 79.7% at three months, and 61.0% at six months), with no significant differences among Kellgren-Lawrence grade II, III, and IV groups (p = 0.538). Adverse drug reactions such as skin rash (n = 23), skin irritation (n = 8), and gastrointestinal disorders (n = 2) were observed. The one-year SFPP use rate was significantly lower in patients in whom these side effects occurred but did not decrease in patients in whom only a skin rash occurred. Overall, 19 patients underwent surgery after discontinuation of SFPP use. Surgery was statistically selected more by the “over 71 years of age” group (p = 0.038) and the “ineffective” group (p = 0.007).

Conclusion: SFPP exerts a comparable therapeutic effect even in end-stage KOA and may be an effective treatment option. Even if patients have end-stage KOA, there are cases in which the patient's background does not allow for surgery positively, such as high perioperative risk or desire for conservative treatment. In such cases, SFPP may be an effective treatment option worth trying.

## Introduction

Musculoskeletal diseases, including osteoporosis and osteoarthritis (OA), lead to a reduction in the mobility of older patients [1]. Among musculoskeletal diseases, knee osteoarthritis (KOA) is regarded as the most common, affecting approximately 4% of the world’s population [2]. In Japan, over 25 million patients aged >40 years have been estimated to suffer from radiographic KOA [3]. Worsening knee joint pain within one year predicts reduced walking ability over a longer follow-up period [1].

The latest Osteoarthritis Research Society International guidelines strongly recommend topical non-steroidal anti-inflammatory drugs (NSAIDs) as a treatment modality for KOA, whereas oral NSAIDs are conditionally recommended [4]. In addition to the existing analgesic medications and formulations, novel treatments have arisen, encompassing new methods, e.g., S-flurbiprofen plaster (SFPP). SFPP has become popular in Japan as a new option for conservative treatment. SFPP (LOQOA® tapes, Taisho Pharmaceutical Holdings Co., Tokyo, Japan) is a new topical NSAIDs patch containing 40 mg of S-flurbiprofen, the active form of racemic flurbiprofen, in a single (40 mg) or two (80 mg) daily application of a tape-type patch [5]. SFPP is not only highly skin permeable but also attains a considerably higher concentration in the synovium than topical flurbiprofen [6]. Systemic exposure to S-flurbiprofen following the application of 80 mg/day of SFPP (two patches/day) for seven days was estimated to be comparable to that of oral formulations of FP [7]; therefore, SFPP was prohibited for patients with gastric ulcer, heavy abnormality of blood, liver, renal, and heart function, similar to oral NSAIDs. Moreover, SFPP should not be used concurrently with oral NSAIDs. In a previous study, clinical symptoms exhibited significant improvement starting at two weeks post SFPP application and improved continuously until 52 weeks after the application [7]. Furthermore, pain relief with SFPP use has been reported to be superior [8]. Several studies have assessed the analgesic efficacy and usefulness of the SFPP; however, all these studies have been conducted on patients with Kellgren-Lawrence (K-L) classification grades II and III (i.e., mild-to-moderate KOA), excluding those with K-L grade IV (i.e., end-stage KOA). To the best of our knowledge, based on our recent literature search, no study has investigated the efficacy and adherence rate of SFPP in patients with K-L grade IV KOA [9].

Therefore, this study aimed to compare the effectiveness and adherence rate of SFPP for pain management among patients with mild-to-moderate and end-stage KOA and to evaluate factors that could relate to the discontinuation of SFPP use in each group.

## Materials and methods

This study was approved by the Institutional Review Board of the author’s affiliated institutions. All patients previously received standard treatment, including oral NSAIDs, acetaminophen, and conventional tape, as outlined in the guidelines [4].

This retrospective cohort study included patients with K-L grades II, III, and IV who regularly visited the outpatient knee clinic at our orthopedic department once a month. The inclusion criteria were as follows: patients with knee pain who understood the numerical rating scale (NRS) for pain, were able to walk, had not taken knee medications within the past month, and were at least 40 years of age. Of all patients who met the inclusion criteria, SFPP was initiated for those who consented after a two-week washout period to minimize the impact of existing drugs in use. Patients with OA secondary to congenital disease, trauma, or infection were excluded from the analysis. Knee pain status was measured using the NRS for pain, with NRS scores ranging from 0 (minimum) to 100 (maximum) [10]. Data on patients’ demographic characteristics, including age, sex, body mass index (BMI), and NRS score at the time of the initial visit, were collected from electronic charts.

The 40 mg SFPP was prescribed and followed up every month to ensure continued use. The dropout rates were assessed after one, three, six, and 12 months. Additionally, whether 50% pain relief and adverse events occurred after SFPP use, as compared with those at the initial visit, was investigated. In this study, “effective” was defined as achieving above 50% pain relief from the initial visit, whereas “ineffective” referred to achieving below 50% pain relief. The percentage of patients who underwent surgical treatment, such as high tibial osteotomy (HTO) and total knee arthroplasty (TKA), was also evaluated.

Statistical analysis

Numerical and categorical variables were presented as means ± standard deviations and as percentages, respectively. Numerical and categorical variables were compared using one-way analysis of variance, Fisher’s exact test, and Pearson’s chi-square test. The statistical significance level was set at P < 0.05. Conditional logistic regression was used to determine the un-adjusted odds ratios (ORs) and 95% confidence intervals (CIs) for adverse events or dropouts. Sociodemographic factors were included as covariates in the adjusted model. As the primary outcome, the overall SFPP use rate at one year was determined. As a secondary outcome, the differences between the dropout and non-dropout groups were determined. The significance level was set at P < 0.05 to be statistically significant. There were 42 cases in the dropout group and 76 cases in the non-dropout group. Therefore, a post hoc calculation (unpaired t-test) of the secondary outcome was performed; a sample size of 0.05 for beta error and 0.8 for effect size was calculated, and G*Power 3.1 (Franz Paul, Kiel, Germany) was used to obtain a beta error of 0.02 (power of 0.98).

## Results

A total of 118 KOA patients (28 males and 90 females; age: 72.3 ± 9.1 years, BMI: 25.3 ± 0.9 kg/m2; NRS score at the first visit: 57.7 ± 11.5) were enrolled between April 2021 and March 2023. Of these patients, 29, 32, and 57 had K-L grade II, III, and IV KOA, respectively. Adverse events were observed in 33 cases (27 patients, 22.9%) during the study period, with skin symptoms being the most prevalent. Of the patients, 23 (19.5%) developed a rash (dermatitis, eczema, erythema, etc.), and eight patients (6.8%) experienced skin irritation. There were no statistically significant differences in the incidence of “adverse events,” “ineffective,” and “undergoing surgery after discontinuation of SFPP use” among the K-L groups. No death or dropout occurred during the follow-up period. The overall SFPP use rate at one year was 61.0% (88.1% at less than one month, 79.7% at three months, and 61.0% at six months). Regarding the one-year SFPP use rate, no differences were observed among the K-L groups (Table 1).

**Table 1:** Patient characteristics NRS: numerical rating scale; TKA: total knee arthroplasty; HTO: high tibial osteotomy; SFPP: S-flurbiprofen plaster.

	Total (n = 118)	K-L grade 2 (n = 29)	K-L grade 3 (n = 32)	K-L grade 4 (n = 57)	P-value
Age	72.3 ± 9.1	70.1 ± 9.9	71.8 ± 8.2	73.7 ± 9.1	0.21
Male	28	4	8	16	
Female	90	25	24	41	
Height	1.56 ± 0.1	1.55 ± 0.1	1.56 ± 0.1	1.57 ± 0.1	0.40
Weight	61.6 ± 6.5	59.9 ± 9.7	61.3 ± 9.3	62.7 ± 13.0	0.53
BMI	25.3 ± 0.9	25.0 ± 4.1	25.3 ± 3.3	25.4 ± 4.5	0.94
Initial NRS	57.7 ± 11.5	57.4 ± 21.3	59.8 ± 23.2	56.8 ± 24.2	0.84
Number of adverse events	33 cases (27 persons)	5	9	19	
Skin rash	23	4	7	12	0.66
Skin irritation	8	0	2	6	0.18
Gastrointestinal disorders	2	1	0	1	0.58
Ineffective	13	2	4	7	0.71
Surgery (TKA or HTO)	19	4	4	11	0.63
Continuing SFPP rate (%)	61.0	63.2	69.0	55.5	0.54

**Figure 1:**
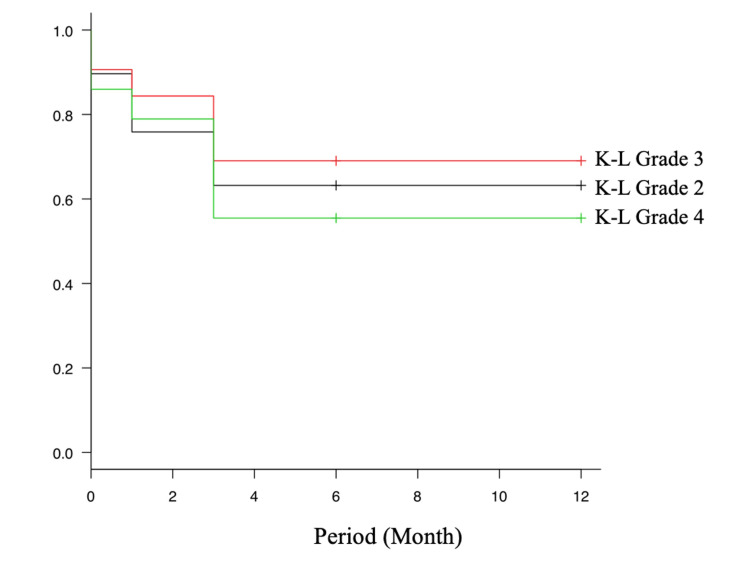
Adherence rates among SFPP users by K-L grade. SFPP: S-flurbiprofen plaster; K-L: Kellgren-Lawrence.

The survival rate of SFPP users according to K-L grades is presented in Figure 1. No statistically significant differences were noted among the groups.

Forty-six (39.0%) patients discontinued SFPP within one year. There were no statistically significant differences between the “dropout group” and the “non-dropout group” in terms of age, sex, BMI, and initial NRS. Drug-induced gastrointestinal disorders occurred in two cases, resulting in the discontinuation of SFPP use. The one-year SFPP use rate did not statically decline in patients who developed any other adverse events. Thirteen patients (11.0%) did not achieve at least 50% initial pain relief, and the one-year SFPP use rate for these patients was 7.7% (OR: 31.2, 95% CI: 4.3-1370.4, P < 0.01) (Table 2).

**Table 2:** Characteristics of patients who did not drop out of SFPP use and those who did SFPP: S-flurbiprofen plaster; NRS: numerical rating scale.

	Total (n = 118)	Non-dropout (n = 76)	Dropout (n = 42)	P-value	Odds	95% CI
Age	72.3 ± 9.1	71.4 ± 9.6	74.0 ± 7.9	0.14		
Male	28	20	8	0.50	0.78	0.28-2.1
Female	90	56	34			
Height	1.56 ± 0.1	1.57 ± 0.1	1.55 ± 0.1	0.16		
Weight	61.6 ± 6.5	61.8 ± 11.4	61.3 ± 11.2	0.83		
BMI	25.3 ± 0.9	25.1 ± 3.9	25.6 ± 4.5	0.49		
Initial NRS	57.7 ± 11.5	59.4 ± 23.1	54.8 ± 23.0	0.30		
Skin rash	23	11	12	0.09	2.2	0.8-6.3
Skin irritation	8	1	7	0.18	14.1	1.7-654.7
Gastrointestinal disorders	2	0	2	<0.01	Inf	0.33-Inf
Rash only	17	10	7	0.60	1.3	0.37-4.1
Ineffective	13	1	12	<0.01	31.2	4.3-1370.4

There were statistically more patients who underwent surgery in the “over 71 years of age” (OR: 3.9, 95% CI: 1.01-22.7, P < 0.01) and “ineffective” (OR: 5.1, 95% CI: 1.3-20.3, P < 0.01) groups (Table 3).

**Table 3:** Characteristics of patients who discontinued SFPP and opted for surgery NRS: numerical rating scale; TKA: total knee arthroplasty; HTO: high tibial osteotomy; SFPP: S-flurbiprofen plaster.

	Non-surgery (n = 99)	Surgery (TKA or HTO) (n = 19)	P-value	Odds ratio	95% CI
Age (>70 years)	57	16	0.040	3.9	1.01-22.7
Age (≤70 years)	42	3			
BMI (>25)	42	12	0.13	2.3	0.76-7.6
BMI (≤25)	57	7			
Effective	91	13	0.010	5.1	1.3-20.3
Ineffective	8	6			
Initial NRS (>60)	50	8	0.62	0.74	0.23-2.2
Initial NRS (≤60)	49	11			
Male sex	24	4	1.00	0.83	0.18-3.0
Female sex	75	15			

In the “ineffective” group, most patients discontinued SFPP use within three months, and 19 patients underwent surgery after discontinuation of SFPP use.

## Discussion

To the best of our knowledge, this is the first study to evaluate adherence to SFPP use in patients with end-stage KOA. Furthermore, this study revealed three significant findings. Firstly, the adherence rate of SFPP was influenced by ineffectiveness and gastrointestinal symptoms. Secondly, adherence rate, adverse events, and achieving pain relief did not differ among K-L grades in this study. Thirdly, a statistically significantly higher number of patients underwent surgery in the “over 71 years of age” and “ineffective” groups.

Ineffectiveness was one of the factors that raised the dropout rate in this study. A phase II, randomized, double-blind, placebo-controlled dose-finding study reported that 72.4% of patients achieved 50% pain relief upon administration of 40 mg of SFPP [11]. In a phase III study, 41.3% (83/201) and 31.8% (64/201) of patients achieved “marked improvement” and “moderate improvement,” respectively, at the end of the study (52 weeks after application or discontinuation). It was also reported that clinical symptoms improved significantly from two weeks after the SFPP application and improved continuously until 52 weeks after the application. Notably, the clinical symptoms exhibited a marked improvement during the initial two months, followed by a gradual improvement [7]. Even among the patients with end-stage KOA in this study, only 10.7% did not attain at least 50% initial pain relief. That indicated that this level of pain relief was expected to be effective even in the end stages of KOA. All dropouts of SFPP use occurred within three months from the start of this study. Based on the findings of both this study and a past study, a period of around three months could serve as a guideline to assess the effectiveness of SFPP for individual patients.

Gastrointestinal disorders were one of the factors that led to the discontinuation of SFPP use in this study. It is well known that oral NSAIDs may cause gastrointestinal adverse events, cardiovascular events, and renal dysfunction. Yataba et al. [7] reported the incidence rate of gastrointestinal adverse events as three out of 101 patients treated with SFPP 40 mg/day and nine out of 100 patients treated with SFPP 80 mg/day over the 52-week treatment period. Only one of the nine patients required additional treatment such as endoscopy or a proton pump inhibitor (PPI). It is worth noting that this patient was also a carrier of *Helicobacter pylori*, which suggests that the adverse event might not necessarily be related to SFPP. In addition, gastrointestinal adverse events were observed at a rate of 2.6% in the SFPP group and 1.9% in the control group, indicating no significant difference between the two groups. As for renal functions, there was no clinically significant increase in blood urea nitrogen or creatinine compared to the baseline after the start of treatment [7,12]. Furthermore, a previous study also reported no cardiovascular complications associated with SFPP. Some patients who developed gastrointestinal symptoms in the study were treated solely by discontinuing SFPP, and they recovered within a week. The incidence of entire adverse events was 22.9% in this study. The incidence of adverse events related to the conditions of the SFPP application site was 10.4% in a phase II study and 5.8% in the diclofenac gel group in the phase III study, with no statistically significant differences, according to the ethical drug package insert. Nevertheless, in the phase III study, most patients (80.1%) completed 52 weeks of SFPP application, despite the occurrence of side effects, including mild dermatitis at the application site, which occurred in 46.8% of patients. They also reported that the major adverse events were “skin symptoms,” which was in accordance with this study. While there were no significant differences in “skin rash” and “skin irritation” between the dropout group and the non-dropout group, but these factors showed a tendency to increase the discontinuation of SFPP use. On the other hand, “rash only” was not a factor for discontinuation. Considering the benefits of SFPP, the frequent occurrence of skin issues does not necessarily imply that patients should discontinue SFPP use.

The one-year SFPP use rate was 61.0% in this study, with half of the participants having end-stage KOA. While this rate was lower than that in the phase III study [7], more than half of the patients were able to continue for one year. In addition, there were no significant differences between K-L grades in terms of side effects, efficacy, or dropout rate. Patients who have undergone successful conservative therapy for over three years have been shown to experience a significantly lower rate of conversion to TKA [13]. SFPP may be a conservative treatment option for patients with end-stage KOA who were more likely to undergo surgery.

Patients who underwent surgery were more inclined to belong to the “over 71 years” and “ineffective” groups. The underlying causes for this trend could not be comprehensively examined within the scope of this study. However, there have been many previous reports on the analgesic effects of surgical treatment [14,15], and there may be a tendency for patients who have reached the limits of conservative treatment and are older to choose surgery to maintain activity.

This study has several limitations. First, this study included and lacked a control group or randomization. As this was an observational study, conservative treatments (e.g., hyaluronic acid and steroid injections, analgesic medications, and physical therapy) prior to the start of SFPP use were not standardized. Furthermore, only 40 mg SFPP was prescribed.

## Conclusions

No significant differences were observed between K-L grades in terms of efficacy and adverse event rates associated with SFPP use, even when considering K-L grade 4. The factors that increased the dropout rate of SFPP use were ineffectiveness and gastrointestinal symptoms. Despite the presence of scattered side effects, such as skin problems, SFPP may be a worthwhile and effective treatment option to consider with high analgesic efficacy and survival rates even for patients with end-stage KOA.
